# The Foot Skin Microbiome in Diabetes Mellitus: Transitional Dysbiosis, Ulceration, and Precision Microbiome-Based Therapeutics: A Narrative Review

**DOI:** 10.3390/jcm15218578

**Published:** 2026-11-03

**Authors:** Iván Julián-Rochina, Mateo López-Moral, Esther García-Morales, Marta Izquierdo-Renau, José David Piñeiro-Ramos, José Luis Lázaro-Martínez

**Affiliations:** 1 Department of Nursing, University of Valencia, 46010 Valencia, Spain; ivan.julian@uv.es (I.J.-R.); marta.izquierdo-renau@uv.es (M.I.-R.); 2 Research Group on Prevention and Treatment of Diabetic Foot Syndrome, University of Valencia, 46010 Valencia, Spain; josedavid.pineiro@universidadeuropea.es; 3 Department of Angiology and Vascular Surgery, La Fe University and Polytechnic Hospital, Avinguda de Fernando Abril Martorell, 106, 46026 Valencia, Spain; 4 Diabetic Foot Unit, Clínica Universitaria de Podología, Facultad de Enfermería, Fisioterapia y Podología, Universidad Complutense de Madrid, Instituto de Investigación Sanitaria del Hospital Clínico San Carlos (IdISSC), 28040 Madrid, Spain; eagarcia@ucm.es (E.G.-M.); diabetes@ucm.es (J.L.L.-M.); 5 Department of Biomedical Sciences, Faculty of Health Sciences, European University of Valencia, Paseo de La Alameda, 7, 46010 Valencia, Spain

**Keywords:** skin microbiome, diabetes mellitus, diabetic foot ulcers, dysbiosis, *Staphylococcus aureus*, microbiome-targeted therapies, bacteriophages, probiotics

## Abstract

Diabetic foot syndrome (DFS) is a severe complication affecting millions of people worldwide, yet its clinical management remains predominantly reactive, focusing on established ulcers rather than early preventive measures. Although the role of the cutaneous microbiome in chronic wounds is recognized, longitudinal ecological shifts in the skin of the foot throughout the progression of diabetes mellitus remain underexplored. This structured narrative review evaluates transitional microbial dynamics from intact skin through to active ulceration and explores emerging microbiome-targeted therapies. Based on a structured literature search of PubMed, Scopus, and Web of Science, complemented by citation searching, and a narrative synthesis of 32 selected publications, current evidence suggests that diabetes-associated changes in the cutaneous microbiome involve heterogeneous alterations in microbial diversity and composition, which vary according to microbial community, anatomical location, and clinical status. DFS progression involves a profound ecological collapse, characterized by depletion of protective commensals (e.g., *Staphylococcus epidermidis*) and the predominance of virulent *Staphylococcus aureus* lineages alongside Gram-negative anaerobes. Consequently, precision interventions such as topical probiotics, synbiotics, and bacteriophage therapy have substantial potential to disrupt recalcitrant biofilms and reprogram host immunity toward pro-regenerative phenotypes. Ultimately, recognizing the skin as a primary target organ in diabetes mellitus highlights the need to shift DFS management toward a proactive framework. Validating these microbiome-modulating therapies through standardized clinical trials is crucial to restoring host-microbiota symbiosis and ultimately preventing lower-extremity amputations.

## 1. Introduction

### 1.1. The Burden of Diabetic Foot Syndrome

Diabetes mellitus (DM) is a major global public health concern affecting more than 529 million people worldwide [1]. Approximately 18.6 million individuals develop diabetic foot syndrome (DFS)-related complications annually [2]. DFS is associated with risk factors including neuropathy, peripheral artery disease, and foot deformities [2], as well as socioeconomic factors [3]. These DFS-related wounds contribute substantially to morbidity, frequently lead to amputation, and impose a substantial economic burden on healthcare systems through the management of chronic wounds [4].

### 1.2. Skin Microbiome and Metabolic Dysbiosis

The skin serves as a vital barrier, harboring a highly complex microbiota predominantly comprising Actinobacteria, Firmicutes, Bacteroidetes, and Proteobacteria, as well as various fungal species [5,6]. This microbiome plays an important role in maintaining cutaneous homeostasis and regulating immune responses; for instance, the commensal *Staphylococcus epidermidis* modulates regulatory T-cell responses and reduces the adhesion of pathogenic species [7], whereas keratinocytes produce antimicrobial peptides [5]. However, metabolic disorders associated with DM exert substantial systemic effects on cutaneous tissues. Hyperglycemia promotes the accumulation of advanced glycation end products (AGEs) and oxidative stress, compromising skin elasticity, impairing keratinocyte differentiation, and reducing antimicrobial peptide production [8]. Collectively, these metabolic alterations disrupt the cutaneous ecosystem and promote dysbiosis [5].

### 1.3. The Diabetic Foot Niche and Chronic Infections

The foot represents a highly heterogeneous microbial niche influenced by intrinsic and environmental factors, including skin thickness and footwear (Table 1) [6]. While healthy foot skin supports a diverse commensal microbiota, the dysbiotic environment of diabetic foot wounds facilitates colonization by opportunistic pathogens, particularly *Staphylococcus aureus* and *Pseudomonas aeruginosa* [4]. These pathogens form complex biofilms that complicate treatment by reducing antimicrobial efficacy and contributing to persistent local inflammation [4]. Furthermore, advances in molecular techniques, such as 16S rRNA gene sequencing, have revealed much greater microbial diversity in these lesions than previously identified using traditional culture-based methods, underscoring the clinical complexity of these infections [9].

### 1.4. Justification and Aims

The skin microbiota associated with diabetic foot ulcers has attracted increasing scientific interest. However, most available research has focused on characterizing the microbiota of established ulcers and evaluating interventions after ulcer onset [4,10,11,12]. To date, no reviews have examined progressive changes in the foot skin microbiome throughout the course of DM, nor have they compared the microbiota of active ulcers with that of adjacent healthy skin from the same individual. A better understanding of these transitional dynamics and of therapeutic strategies aimed at modulating the microbiome is crucial for developing preventive clinical strategies for DFS. To address this gap, this narrative review aims to answer the following questions: (i) What is the current evidence regarding changes in the skin microbiome in individuals with DM? (ii) How are these alterations associated with the subsequent onset of DFS? (iii) Which diagnostic techniques are useful for detecting imbalances in the skin microbiome? (iv) What nonpharmacological treatments may help restore the skin microbiota in individuals with DM?

## 2. Methods

### 2.1. Search Strategy and Temporal Justification

This study was designed as a structured narrative review to examine DM-associated changes in the foot skin microbiota and their potential role in the onset of DFS. A structured literature search was conducted in PubMed, Scopus, and Web of Science to identify relevant publications. Given the broad, exploratory objectives of the review and the heterogeneity of the available evidence, findings were integrated through a thematic narrative synthesis. The review was not designed as a systematic review, and no meta-analysis was performed. The primary electronic database search was restricted to articles published from January 2020 through August 2026. This timeframe was selected based on a bibliometric analysis of the targeted search query, incorporating terms such as “microbiome,” “skin,” and “diabetic foot”. Historically, the field has undergone significant paradigm shifts: research before 2010 focused almost exclusively on traditional bacterial culture methods and antibiotic resistance, whereas the period from 2011 to 2019 saw the preliminary introduction of microbiome concepts. Notably, approximately 60% of the total scientific literature meeting these specific search criteria was published between 2020 and 2026. This accelerated expansion reflects a shift in the research landscape, marked by the increasing application of metagenomic sequencing, a growing understanding of biofilm dynamics, and the emergence of microbiome-modulating therapies. Restricting the search to this highly productive period was intended to ensure that the review captured this systematic understanding of host-microbiome interactions rather than focusing primarily on earlier culture-based approaches. The exact search strings, keywords, and Boolean operators used for each database are provided in Supplementary Material S1.

**Figure 1:**
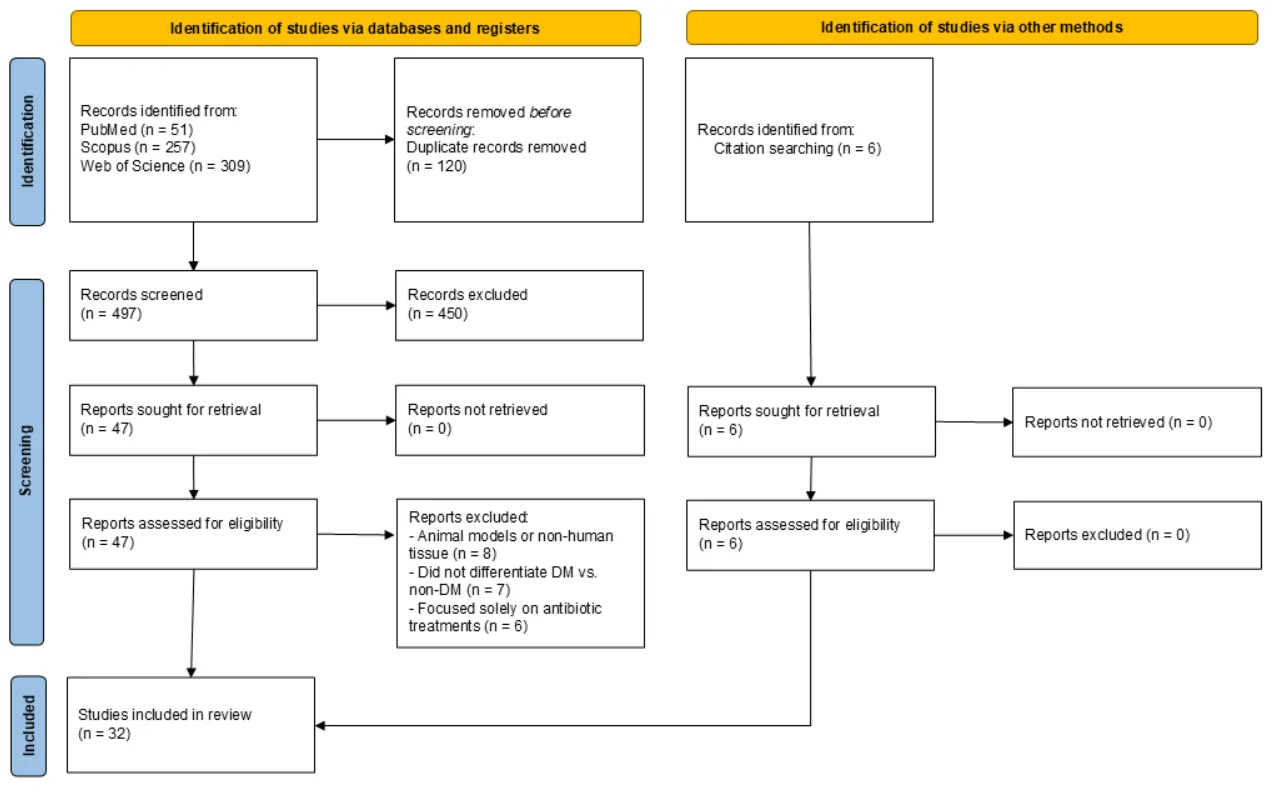
Flow diagram documenting the literature identification and selection process using the PRISMA 2020 format.

To complement the primary database search, backward citation searching was performed by examining the reference lists of relevant publications. This process identified six additional articles published before 2020 that were repeatedly cited in the retrieved literature and considered relevant to the methodological or clinical objectives of the review: Chakravorty et al. (2007) [13], Gontcharova et al. (2010) [14], Oates et al. (2012) [15], Redel et al. (2013) [16], Yang et al. (2016) [17], and Gardiner et al. (2017) [18]. These six publications correspond to the records identified through citation searching in Figure 1.

The primary database search was specifically designed to identify publications concerning the foot skin microbiome in diabetes mellitus and diabetic foot disease. Given the integrative nature of this narrative review, related topics, including the mycobiome, bacteriophages, probiotics, fecal microbiota transplantation, CRISPR-based approaches, nanotechnology, and multi-omics, were also discussed using relevant publications identified during the review process, particularly narrative and other literature reviews and the evidence synthesized therein. No separate systematic searches were conducted for each of these specific topics, as the objective was to provide a broad thematic overview rather than an exhaustive assessment of individual diagnostic or therapeutic strategies.

### 2.2. Inclusion and Exclusion Criteria

After duplicate records were removed, articles were screened based on predefined criteria. Articles were eligible for inclusion if they met at least two of the following criteria: (1) skin microbiota; (2) populations with DM; (3) diabetic foot; (4) pathogens commonly associated with the diabetic foot; or (5) treatments that modulate the skin microbiota. For primary human studies contributing to the core comparative evidence, the following exclusion criteria were applied: (1) studies conducted in animal models or using nonhuman tissues; (2) studies that did not differentiate results according to the presence or absence of DM; (3) studies focusing exclusively on the wound bed without considering perilesional or healthy skin as an ecological comparator; (4) articles focused exclusively on antibiotic treatment without evaluating microbiota-modulating therapies for comparison; and (5) articles written in non-English/non-European languages.

It should be noted that the inclusion and exclusion criteria were primarily intended to guide the selection of studies addressing the main clinical and microbiological objectives of the review. Given its narrative scope, additional publications, including relevant literature reviews, preclinical investigations, and studies focusing on established wounds, were also cited when they provided valuable mechanistic, methodological, or therapeutic context. These complementary references were used to support the narrative discussion and were not considered equivalent to primary comparative clinical evidence.

### 2.3. Study Selection and Narrative Review Quality Framework

To promote transparency and minimize selection bias, two independent reviewers screened the titles and abstracts of the initially retrieved records. The full texts of potentially relevant articles were then assessed for eligibility. Any disagreements between the two reviewers regarding eligibility were resolved through discussion and consensus, with a third senior reviewer consulted when necessary.

The Scale for the Assessment of Narrative Review Articles (SANRA) was consulted as a guiding framework to support the overall quality and reporting of the narrative review manuscript [19,20]. SANRA was not used to assess the methodological quality of individual publications. No formal study-level methodological quality or risk-of-bias assessment was performed.

The literature identification and study-selection process is presented in a flow diagram using the PRISMA 2020 format (Figure 1) [21]. This diagram is intended to transparently document the search and selection procedures and does not imply that the review was conducted as a systematic review.

The 32 selected publications contributing to the thematic narrative synthesis, including original research articles and supporting methodological and review literature, are categorized according to their primary thematic domain and publication type in Supplementary Material S2.

During the preparation of this manuscript, the authors used Gemini (Google) for language polishing, formatting the reference list according to the journal’s style, and structural editing to condense the abstract to meet the required word limit. The AI tool was not used for study design, literature selection, data collection, analysis, interpretation, or the generation of scientific conclusions. The authors have critically reviewed and edited all outputs and take full responsibility for the final content of the manuscript.

## 3. Results

### 3.1. Molecular Diagnostic Techniques: Overcoming the Limitations of Traditional Cultures

Traditional culture-based methods substantially underestimate microbial diversity in diabetic foot syndrome (DFS) and frequently fail to detect slow-growing, unculturable, or biofilm-embedded pathogens, as well as obligate anaerobes. Amplicon sequencing (e.g., 16S rRNA gene sequencing) helps overcome this limitation by enabling broad microbial taxonomic profiling [22,23]. However, its clinical application in DFS remains subject to significant translational barriers. The selection of specific 16S rRNA hypervariable regions (V1–V9) introduces notable taxonomic biases [13,17]. While the V4–V6 regions are generally recommended for cutaneous microbiota, many DFS studies use the V3–V4 region—a convention largely derived from research on the gut microbiome—often without adequate methodological justification, highlighting the need for standardized protocols for skin sampling [11,17,24,25,26,27]. Furthermore, 16S rRNA sequencing provides limited functional information on antibiotic resistance genes (ARGs) and virulence factors and does not capture bacteriophages and eukaryotic viruses that significantly influence the virulence and biofilm-forming capacity of *Staphylococcus aureus* [22,23].

To address these limitations, metagenomic next-generation sequencing (mNGS) has emerged as a promising clinical tool. By fragmenting and sequencing DNA extracted from a sample, mNGS provides species-level and, in some cases, strain-level resolution and enables functional profiling of ARGs and virulence factors, which directly informs targeted antimicrobial therapy and prediction of clinical outcomes [22,28]. Despite these diagnostic advantages, routine implementation of mNGS in DFS management remains limited by high costs, the need for complex bioinformatics infrastructure, substantial human DNA contamination in wound samples, and the inability to reliably distinguish between viable, infection-causative microorganisms and nonviable extracellular DNA [23,28]. Furthermore, important translational challenges remain in the clinical interpretation of mNGS datasets; DNA-based sequencing detects the presence of resistance and virulence genes but does not directly quantify their expression levels, and the complexity of multi-omics reports poses a major barrier to clinicians attempting to make rapid, actionable antibiotic selections in routine practice [22].

As an adjunct to molecular sequencing, real-time metabolic biomarkers (e.g., pH, uric acid, pyocyanin, and L-tyrosine), alongside specific immunological and proteolytic markers such as the IL-1β/IL-1RA ratio and elevated MMP-9 levels, are being explored for monitoring the wound microenvironment and distinguishing different healing trajectories [11,29,30]. Furthermore, to better capture the true functional activity of the wound bed rather than microbial presence alone, diagnostics should expand beyond DNA-based mNGS to include metatranscriptomics and metaproteomics [30]. As highlighted by recent comparative multi-omic analyses, this evolution is clinically imperative because microbial DNA abundance does not necessarily correlate with metabolic activity; highly active causative pathogens often exist at very low relative abundances, potentially limiting the utility of DNA-based compositional profiles in guiding targeted therapy [28]. These multimodal indicators have been associated with infection status and wound progression, supporting the development of emerging smart wound dressings equipped with nanosensors designed to dynamically release therapeutics [31]. Furthermore, the integration of artificial intelligence (AI) and machine learning algorithms with multi-omic datasets is increasingly being used to transform these diagnostic readouts into predictive tools for biofilm formation and personalized therapeutic responses [31,32].

### 3.2. Systemic Metabolic Disruption and the Pathogenesis of Cutaneous Dysbiosis

#### 3.2.1. Loss of Homeostasis and Metabolic Inflammation

Under physiological conditions, the commensal skin microbiota actively contributes to maintaining cutaneous homeostasis; for example, *Staphylococcus epidermidis* produces antimicrobial compounds, modulates regulatory T-cell activation, and attenuates local inflammation through TLR2 signaling, thereby limiting the proliferation of pathogens such as *Staphylococcus aureus* [5,23,33]. In diabetes mellitus (DM), this homeostatic balance is severely disrupted by systemic metabolic derangements. Hyperglycemia and insulin resistance promote a pro-inflammatory state characterized by elevated levels of IL-6 and TNF-α, which can impair keratinocyte function and alter the lipid composition of sebum [8]. Furthermore, activation of the AGE-RAGE pathway promotes the generation of reactive oxygen species (ROS) and contributes to extracellular matrix degradation, while impaired phagocyte metabolism leaves excess extracellular glucose, which directly enhances pathogen virulence [8,34].

#### 3.2.2. The Virulence and Adaptability of *Staphylococcus aureus*

This metabolically compromised, glucose-rich microenvironment strongly favors the proliferation of *S. aureus*, the predominant colonizer in DFS-associated lesions [34]. Through the use of alternative sigma factors and global regulators, *S. aureus* exhibits remarkable metabolic plasticity, adapting to cutaneous stressors, host immune responses, and local hypoxia [18,34]. The pathogen secretes proteases, hemolysins, and collagenases that facilitate tissue invasion and biofilm formation, thereby complicating microbial eradication [34].

Beyond their role in direct tissue degradation, these secreted proteases actively subvert host immunity. Specifically, staphopain B (SspB) selectively cleaves phagocytic receptors (e.g., CD11b) to induce targeted apoptosis in macrophages and dendritic cells, thereby impairing bacterial clearance mechanisms and maintaining the wound in a state of persistent chronic inflammation [35]. Furthermore, *S. aureus* secretes the epidermal cell differentiation inhibitor (EDIN) toxin, which actively disrupts epithelial and endothelial cohesion, facilitating pathogen dissemination into deeper ulcer tissues while simultaneously inducing host cell death through inflammatory cell-death mechanisms such as pyroptosis and necroptosis [35].

Additionally, *S. aureus* establishes a protected intracellular niche within the epidermis by actively suppressing the host expression of Perforin-2 (P2)—an antimicrobial protein essential for clearing intracellular bacteria—which further exacerbates inflammasome activation and impairs wound healing [36]. Beyond simply residing within host cells, the harsh, hyperglycemic, and hypoxic microenvironment of the DFU actively drives *S. aureus* to undergo profound phenotypic switching to Small Colony Variants (SCVs). These dormant, slow-growing SCVs drastically downregulate major virulence factors to evade immune detection while establishing highly recalcitrant intracellular reservoirs that withstand aggressive antibiotic therapies, ultimately contributing to the chronicity of infection and recurrent infection cycles characteristic of advanced diabetic wounds [34]. Notably, the high clonal diversity of these isolates (e.g., CC45, CC30, and CC398) indicates that patients with DM serve as critical reservoirs for highly virulent and multidrug-resistant lineages, underscoring the urgent need for stringent epidemiological surveillance in clinical settings [18].

**Figure 2:**
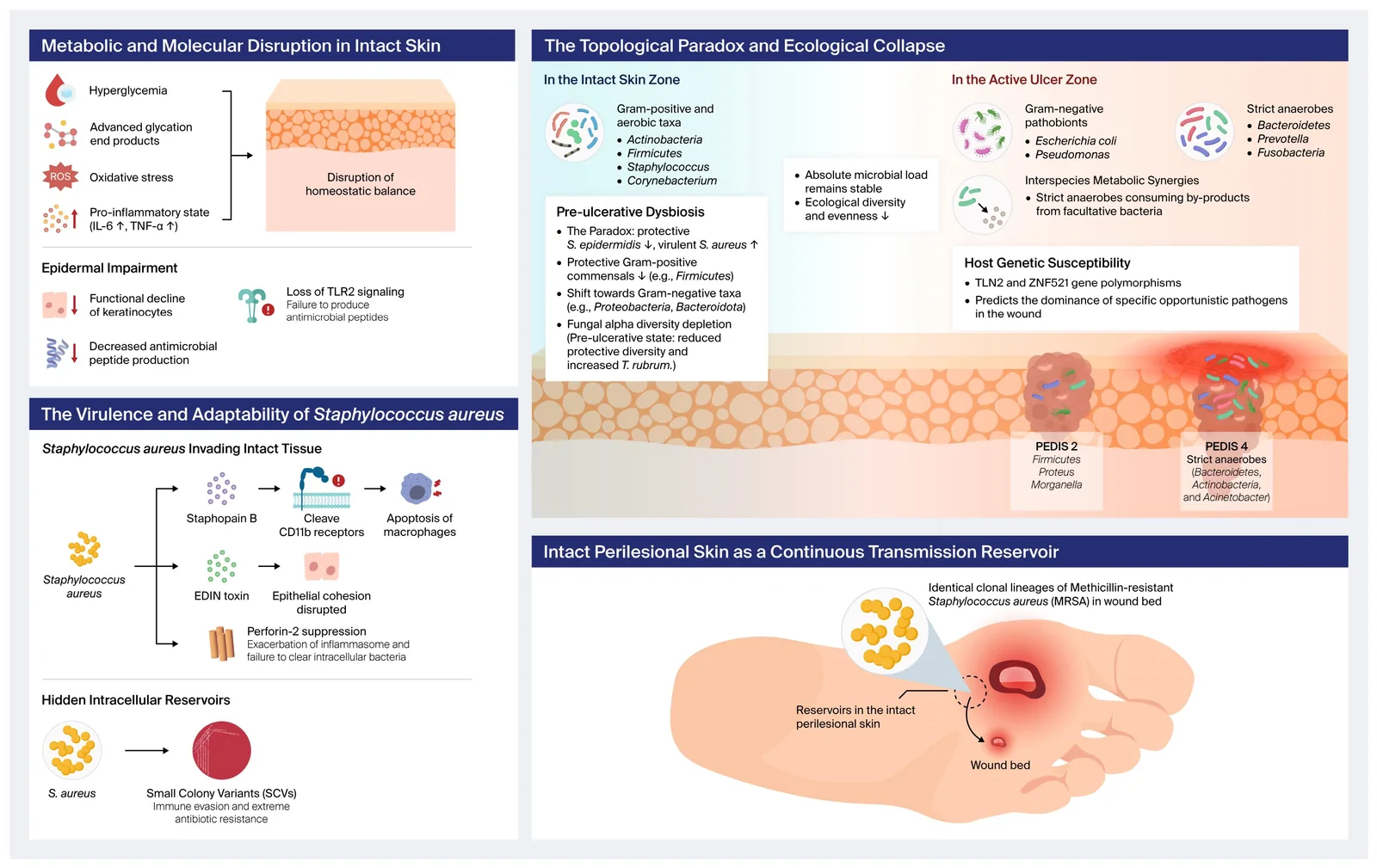
Conceptual model of microbiome-related mechanisms in diabetic foot disease. The diagram integrates findings from heterogeneous observational and experimental studies, illustrating biologically plausible associations rather than an established temporal sequence or causal pathway. (**Left**) Potential effects of diabetes-associated metabolic alterations on epidermal barrier function and microbial pathogenicity. (**Right**, upper) The term “topological paradox” is used descriptively to highlight apparently contrasting patterns of microbial richness, diversity, evenness, and community composition across intact skin, pre-ulcerative states, and established diabetic foot ulcers. These differences may reflect variations in anatomical location, clinical characteristics, and methodological approaches and should not be interpreted as a universal ecological progression. (**Right**, lower) Hypothesized role of intact perilesional skin as a potential microbial reservoir. Although microbial similarities between intact skin and ulcers support this hypothesis, the direction of microbial transmission has not been established.

The molecular disruption of intact skin, including the loss of TLR2 signaling and the intracellular invasion mechanisms of *S. aureus* described above, are visually summarized in Figure 2 (left section).

#### 3.2.3. Clinical Evidence of Ecological Shift

Cross-sectional molecular studies comparing individuals with DM with healthy controls have reported changes in microbial diversity and community composition, although the direction and magnitude of these alterations vary across studies and microbial communities [16,18,24].

Interestingly, longitudinal 16S rRNA profiling reveals that the most abundant cutaneous taxa often remain compositionally similar in healthy and diabetic individuals; the dysbiotic signature appears to be primarily driven by the marked depletion of the “rare biosphere” (low-abundance commensal taxa). Notably, the application of Random Forest machine learning algorithms has demonstrated that this specific loss of rare taxa can accurately predict an individual’s diabetic status with high sensitivity even before ulceration occurs [18]. Crucially, these pre-ulcerative ecological shifts disproportionately impact the cutaneous mycobiome; while overall bacterial community structures may initially remain relatively stable on intact diabetic skin, fungal communities undergo significant compositional divergence and a marked loss of alpha diversity early in the disease process [24].

As the disease advances, the bacterial microbiome also undergoes dynamic remodeling; notably, high-throughput sequencing of intact diabetic skin has revealed a progressive increase in bacterial alpha diversity and richness with increasing disease duration, suggesting an initial adaptive and compensatory response to the hyperglycemic microenvironment [37]. It is important to distinguish species richness (the number of taxa), evenness (their relative distribution), alpha diversity (within-sample diversity measured using indices that may weight richness and evenness differently), and temporal stability (changes in microbial communities over time). Therefore, increased richness or changes in taxonomic composition should not necessarily be interpreted as indicating a healthier or more stable microbial ecosystem. However, as the clinical risk of ulceration increases, as reflected in the International Working Group on the Diabetic Foot (IWGDF) risk stratification system, this adaptive pre-ulcerative state ultimately gives way to the pronounced pathogenic dysbiosis seen in active lesions [38]. Higher-risk categories are associated with greater depletion of protective Gram-positive commensals (Firmicutes, including *S. epidermidis*) and an overrepresentation of aggressive Gram-negative taxa (Proteobacteria and Bacteroidetes), alongside pathogenic fungi such as *Trichophyton rubrum* [24,38]. Furthermore, deep metagenomic sequencing has identified specific Gram-negative opportunistic pathogens, such as *Moraxella osloensis*, as core predictive biomarkers for high-risk pre-ulcerative states in human cohorts [38]. Moreover, individual susceptibility to this ecological collapse is profoundly influenced by host genetics; specific host polymorphisms (e.g., in the *TLN2* and *ZNF521* genes) have been shown to explain up to 53% of the variation in microbial diversity and strongly predict the dominance of specific opportunistic pathogens [30].

The specific demographic characteristics, methodological details, and taxonomic findings of these foundational studies are summarized in Table 2.

### 3.3. Microbial Signatures of DFS: From Intact Skin to Active Ulceration

#### 3.3.1. The Collapse of Ecological Diversity

Comparative analyses of the skin microbiota of healthy individuals and the microbiota of DFS-associated wounds reveal a consistent pattern of ecological collapse upon ulceration. While intact, healthy skin is predominantly colonized by Gram-positive and aerobic taxa (such as Actinobacteria, Firmicutes, *Staphylococcus*, and *Corynebacterium*), the microenvironment of the diabetic ulcer undergoes profound ecological restructuring. Interestingly, some studies report that the ulcer bed can exhibit a higher number of unique taxa (richness) than intact skin, likely due to colonization by transient environmental microbes and opportunistic pathogens [39]. However, despite this influx of new taxa, overall ecological diversity and community evenness often collapse; the wound microenvironment becomes overwhelmingly dominated by a few highly virulent pathogens, disrupting the protective balance maintained by commensal microorganisms [39]. Importantly, foundational studies employing quantitative PCR (qPCR) alongside high-throughput sequencing have demonstrated that the absolute microbial load (total bacterial and fungal counts) often remains comparable between healthy and intact diabetic skin; the primary pathological shift lies not in mere microbial overgrowth but in this qualitative restructuring and dominance within the community [16].

The wound bed consistently demonstrates a structural shift toward Gram-negative and obligate anaerobic bacteria, with marked enrichment of phyla such as Proteobacteria, Bacteroidetes, and Fusobacteria, and genera including *Prevotella*, *Bacteroides*, and *Proteus* [7,14,15,18,39,40,41,42]. Crucially, comparative 16S rRNA analyses across healthy, pre-ulcerative diabetic, and active DFU cohorts reveal a distinct and contrasting shift in microbial composition: while commensal *Staphylococcus* often dominates and its relative abundance increases on the intact skin of individuals with diabetes, its abundance declines sharply within the ulcer bed, where opportunistic Gram-negative pathobionts such as *Escherichia coli* and *Pseudomonas* become more prominent [40]. Recent shotgun metagenomic profiling across clinical severity grades reveals that this anaerobic shift is tightly coupled to disease progression; while moderate infections (PEDIS grades 2–3) are predominantly driven by facultative Proteobacteria, severe anoxic lesions (PEDIS grade 4) exhibit a profound collapse in overall microbial diversity and are overwhelmingly dominated by obligate anaerobes (Bacteroidetes), creating resilient polymicrobial communities that severely impair tissue oxygenation and healing [42]. This ecological collapse extends beyond bacterial populations; targeted high-resolution profiling reveals that fungal alpha diversity is also significantly diminished in chronic DFUs, a shift accompanied by enrichment of intrinsically multidrug-resistant fungal pathogens, such as *Candida duobushaemulonii*, which severely complicate standard antimicrobial management [43]. Furthermore, initial microbial composition serves as a potent predictive biomarker of long-term wound outcomes; for example, elevated baseline abundance of Gram-positive anaerobic cocci (GPAC)—most notably the genus *Peptoniphilus*—has been statistically associated with impaired healing trajectories and clinical failure at 4 weeks, underscoring the prognostic value of early microbiome profiling [39].

As complementary longitudinal evidence from studies focusing on established diabetic foot ulcers without an intact-skin comparator, Sloan et al. (2019) [44] demonstrated that microbial diversity is not a static parameter but fluctuates dynamically with clinical events. For instance, ecological diversity decreases sharply during acute clinical infections—often driven by the rapid expansion of specific taxa such as *Staphylococcus* or *Streptococcus*—but typically recovers upon clinical resolution [44]. Furthermore, nonhealing DFUs are characterized by highly stable, heterogeneous communities dominated by strict anaerobes and Enterobacteriaceae [44]. The persistence of these climax communities is reinforced by intricate interspecies metabolic synergies; network modeling indicates that strict anaerobes (e.g., *Porphyromonas asaccharolytica*) derive essential metabolic by-products from facultative Gram-negative bacteria (e.g., *Escherichia coli*), creating a resilient microenvironment that actively resists host clearance [44].

#### 3.3.2. Pathogen Persistence and Intraindividual Transmission

Beyond broad taxonomic shifts, the persistence of specific pathogenic lineages underscores the clinical challenge associated with DFS. *Staphylococcus aureus*—particularly methicillin-resistant strains (MRSA)—remains the most frequently isolated pathogen in active ulcers [15,45]. Crucially, genomic profiling reveals that *S. aureus* isolates recovered from both the healthy, intact skin and active ulcer of the same patient often belong to the same clonal lineages and share highly similar virulence and resistance genotypes [45]. This potential for intraindividual transmission is further supported by spatial microbiome mapping, which reveals no significant compositional differences between the active ulcer bed and the immediate perilesional intact tissue. This ecological similarity is consistent with the hypothesis that intact perilesional skin may act as a potential microbial reservoir. However, the available observational evidence does not establish the direction of microbial transmission or exclude colonization of both sites from a common source [41,45]. Consequently, these findings support the rationale for investigating comprehensive skin decontamination and microbiome modulation strategies targeting the broader lower extremity before ulceration occurs, rather than focusing solely on the localized wound bed.

These findings are integrated into the conceptual framework presented in Figure 2. The term “topological paradox” is used descriptively to illustrate the apparently contrasting patterns of microbial richness, diversity, and community composition observed across different anatomical sites and clinical stages of diabetic foot disease. These patterns reflect heterogeneous evidence and should not be interpreted as a universally established sequential or causal pathway from intact skin dysbiosis to ulceration.

#### 3.3.3. Methodological Heterogeneity and Translational Barriers

Despite the clear overarching trends, the current literature is severely limited by methodological heterogeneity. The reviewed studies exhibit substantial differences in inclusion criteria, anatomical sampling sites (e.g., plantar arch vs. heel or tibia), and sampling protocols (superficial swabs vs. deep tissue biopsies) [14,15,39,41]. Importantly, recent comparative molecular analyses have addressed this specific methodological debate; evidence confirms that rigorous superficial sampling—specifically using the Levine swabbing technique to sample deeper wound fluid—can achieve high microbial concordance with invasive deep tissue biopsies, supporting the diagnostic utility of this noninvasive approach for DFI microbiome profiling [7]. The anatomical location represents a major confounding variable; recent evidence demonstrates that as ulcers progress toward healing, the recovering microbiome differs significantly according to local skin physiology, with moist plantar regions selecting for microbial communities that differ from those of dry lower-leg regions [41]. Furthermore, the lack of standardization in sequencing approaches—ranging from analyses targeting different hypervariable regions (V1–V2, V3–V4) to whole-genome sequencing (WGS) and metagenomic next-generation sequencing (mNGS)—limits comparability across studies [7,18,40,42,45]. To advance clinical management, future research must adopt standardized sampling frameworks that consistently assess both the wound bed and the perilesional intact skin as an ecological comparator.

The specific demographic characteristics, inclusion/exclusion criteria, and microbiological findings of these core comparative studies are summarized in Table 3.

### 3.4. Microbiota-Targeted Therapies: Beyond Conventional Antimicrobials

The therapeutic approaches discussed in this section are supported by heterogeneous levels of evidence, ranging from preliminary human clinical findings to preclinical, in vitro, and experimental investigations. Consequently, these emerging strategies should be considered according to their respective stage of development, and promising mechanistic findings should not be interpreted as established clinical efficacy or safety in patients with diabetic foot disease.

#### 3.4.1. The Gut-Skin Axis and Systemic Modulation

Hyperglycemia severely compromises intestinal barrier integrity, leading to systemic dysbiosis characterized by a reduction in protective flora (e.g., *Bifidobacterium* and *Lactobacillus*) and an overgrowth of opportunistic pathogens [46,47]. This intestinal imbalance alters the production and systemic translocation of crucial microbiota-derived metabolites. While the depletion of short-chain fatty acids (SCFAs) deprives the host of important anti-inflammatory effects, the concurrent systemic accumulation of gut-derived endotoxins—specifically lipopolysaccharides (LPS) and trimethylamine-N-oxide (TMAO)—actively disrupts cutaneous homeostasis. These noxious metabolites trigger endoplasmic reticulum (ER) stress and widespread Toll-like receptor 4 (TLR4) activation, which directly impairs tissue repair by disrupting the essential polarization of wound macrophages toward the reparative M2 phenotype [46]. Emerging evidence highlights that this axis operates bidirectionally; chronic dermal injury in DFUs drives a “skin-to-gut axis” that disrupts intestinal immune homeostasis, creating a vicious cycle that perpetuates systemic inflammation and impairs healing [48]. Furthermore, systemic microbial regulation is driven not only by SCFAs—which enhance skin barrier repair via histone deacetylase (HDAC) inhibition and the activation of PPAR-γ and aryl hydrocarbon receptors (AhR)—but also by tryptophan metabolites (e.g., kynurenine and indole derivatives), which profoundly modulate local immune cell function [48].

Probiotic and synbiotic supplementation has been investigated for its potential effects on microbial balance and inflammatory markers. However, clinical evidence supporting its efficacy specifically in the management of diabetic foot ulcers remains limited [47,49]. In parallel, targeted nutraceutical interventions, such as the administration of the bioactive flavonoid quercetin, have recently been shown to remodel the gut microbiota, specifically enriching anti-inflammatory taxa such as *Ligilactobacillus* while reducing pro-inflammatory *Desulfovibrionia*, with associated stabilization of HIF1α and upregulation of VEGF in the wound bed, thereby accelerating wound closure. Furthermore, emerging systemic strategies such as fecal microbiota transplantation (FMT) have shown promising results in preclinical animal models, successfully promoting local angiogenesis, restoring intestinal barrier integrity (e.g., upregulating ZO-1 and Occludin), and accelerating wound closure via the IL-17A-mTOR-HIF1α signaling axis, although robust human trials are still needed before clinical translation [32,48,50]. Crucially, the ultimate therapeutic target of these microbiome-directed interventions—whether achieved through systemic synbiotics or topical probiotics—is the reprogramming of host innate immunity. Preclinical and mechanistic studies suggest that microbiome-directed interventions may influence macrophage polarization toward reparative phenotypes and modulate growth-factor signaling pathways involved in tissue repair. However, these mechanisms have not yet been established as clinical benefits in patients with diabetic foot ulcers [47]. At the systemic level, these interventions promote innate immune cell chemotaxis, shift macrophages toward anti-inflammatory M2 phenotypes, and stimulate angiogenesis, thereby contributing to the restoration of a metabolic environment conducive to chronic wound healing [47,51].

#### 3.4.2. Topical Probiotics and Commensal Stimulation

Applying probiotics directly to the wound bed offers a highly targeted ecological intervention. Strains such as *Lactiplantibacillus plantarum* and genetically engineered *Limosilactobacillus reuteri*—the latter specifically modified to express therapeutic chemokines such as CXCL12-α directly within the wound microenvironment—have demonstrated potential to reduce pathogenic bacterial loads, attenuate biofilm formation, and accelerate re-epithelialization [5,46,47]. To maximize these effects, recent research has shifted from isolated probiotic application toward the use of synbiotics—formulations that combine live beneficial microbes with targeted prebiotics (e.g., nondigestible oligosaccharides). Prebiotics actively nourish the applied probiotics, significantly extending their viability in the harsh wound microenvironment while synergistically enhancing their immunomodulatory and tissue-regenerative properties [49]. However, the clinical translation of these advanced, engineered microbiome-based therapies (MBTs) necessitates stringent biocontainment strategies; ongoing research is evaluating mechanisms such as stable genetic “kill switches” and auxotrophy (which requires an exogenous nutrient) to selectively terminate the engineered bacteria, thereby limiting uncontrolled proliferation and mitigating the risk of environmental cross-contamination [5]. Recent investigations have expanded this therapeutic repertoire to include species such as *Bacillus clausii* and *Bacillus coagulans*, emphasizing that localized topical delivery can minimize systemic exposure and potentially mitigate associated risks in vulnerable diabetic populations [52].

Concurrently, therapies aimed at stimulating indigenous skin commensals (such as *Staphylococcus epidermidis* and *Cutibacterium acnes*) are gaining traction. Mechanistically, these commensals actively antagonize pathogen virulence; for example, *S. epidermidis* secretes serine proteases (Esp) that degrade *S. aureus* biofilm adhesion proteins (FnBPA and Spa) and produces autoinducing peptides that potently suppress the *S. aureus* accessory gene regulator (Agr) quorum-sensing system [23]. Beyond direct microbial competition, these commensals act as critical immune adjuvants; species such as *S. epidermidis* secrete small molecules that activate Toll-like receptor 2 (TLR2) signaling on host keratinocytes, driving the production of endogenous antimicrobial peptides (AMPs), such as cathelicidin (LL-37) and human β-defensins, which can disrupt *S. aureus* membranes [51]. Furthermore, commensal lipid metabolism generates free fatty acids, such as sapienic acid, which maintain the skin’s acidic mantle and exert direct, selective bactericidal activity against pathogenic *S. aureus* strains while sparing resident flora [51]. By enhancing these intrinsic antibacterial defenses against methicillin-resistant *S. aureus* (MRSA) and downregulating local tissue inflammation, commensal modulation represents a critical pathway for restoring the cutaneous microbial balance [47,49].

Importantly, the effects of skin commensals extend beyond ecological competition; they also actively support host immune function; they promote epidermal barrier repair and integrity through activation of the aryl hydrocarbon receptor (AhR) in keratinocytes and the modulation of T-lymphocyte subsets involved in tissue repair [36]. Moreover, these commensals orchestrate an innate immune repair cascade by initiating a type I interferon (IFN) response via dendritic cell recruitment, which subsequently stimulates fibroblasts and macrophages to release essential proregenerative growth factors [36]. Beyond ecological replacement, the therapeutic horizon is shifting toward precision microbiome editing via CRISPR-Cas systems [31]. Experimental CRISPR-Cas approaches are being investigated for the selective targeting of antibiotic resistance and virulence genes in pathogens such as *S. aureus*, although their clinical application in diabetic foot disease remains unproven [31].

#### 3.4.3. Advanced Delivery Systems: Nanotechnology and Vesicles

To overcome the highly proteolytic and hostile microenvironment of diabetic foot ulcers (DFUs), advanced delivery systems are essential. Probiotic encapsulation within biocompatible matrices (such as chitosan, alginate, and hydrogels) protects microbial viability, ensures controlled release, and improves tissue adhesion at the site of infection [46,47]. Emerging bioengineered platforms, including electrospun nanofiber scaffolds, function simultaneously as protective barriers and sustained-release systems; however, the clinical translation of viable microorganism-based therapies requires particular caution, as the theoretical risk of bacterial translocation in severely ischemic or immunocompromised DFUs remains insufficiently characterized in large randomized controlled trials [52]. Furthermore, cell-free therapies utilizing probiotic-derived biogenic nanoparticles (incorporating elements such as silver, selenium, or copper) and extracellular vesicles represent an emerging therapeutic approach. These nanoscale systems deliver potent antibiofilm, antimicrobial, and antioxidant properties deep into the tissue while potentially reducing the systemic risks associated with live bacterial administration in compromised patients [46,47,49].

#### 3.4.4. Bacteriophage Therapy for Multidrug-Resistant Pathogens

The escalating crisis of antimicrobial resistance in DFS necessitates alternative eradication strategies. Phage therapy, particularly targeting *S. aureus*, is being investigated as a potential adjunctive strategy against difficult-to-treat diabetic foot infections [53]. Unlike conventional antibiotics, bacteriophages offer a “surgical strike” advantage with self-amplifying properties at the site of infection, utilizing phage-derived depolymerases to actively degrade the biofilm-associated extracellular polymeric substance (EPS) matrix and restore bacterial susceptibility to host defenses [31]. Early clinical reports and exploratory investigations have described encouraging outcomes with customized bacteriophage preparations, either alone or in combination with conventional antibiotics. However, current evidence remains insufficient to establish their efficacy in promoting ulcer healing or preventing amputations [53]. Despite these highly promising outcomes, the translation of phage therapy into routine clinical practice faces significant methodological and regulatory hurdles. Current preclinical evidence relies heavily on acute wound models in type 1 diabetes, which do not accurately recapitulate the complex, chronic pathophysiology of type 2 diabetes that is most prevalent in human DFIs [53]. Furthermore, while early-phase clinical trials (such as the REVERSE and DANCE trials) support short-term safety, rigorous phase III studies are urgently needed to establish standardized dosing protocols and evaluate long-term risks, including the emergence of phage resistance and potential neutralization of phages by host immune responses, including antiphage antibodies [53].

#### 3.4.5. Targeted Metabolic Reprogramming

Beyond ecological replacement and mechanical eradication, emerging therapeutic paradigms directly target the metabolic adaptability of *S. aureus*. Given that the hyperglycemic microenvironment of the DFU actively fuels bacterial glycolysis and virulence expression, novel compounds designed to specifically inhibit glycolysis (such as 2-deoxyglucose formulations) have demonstrated the ability to essentially “starve” the pathogen, significantly reducing bacterial load and biofilm formation in preclinical models [33]. Furthermore, by pharmacologically inhibiting key bacterial metabolic nodes, such as the tricarboxylic acid (TCA) cycle or amino acid transport systems (e.g., the Catabolite Control Protein A, CcpA), these approaches can drastically attenuate virulence factor secretion and reverse antibiotic resistance in MRSA strains, offering a potent adjunctive strategy to conventional antimicrobial regimens [33].

### 3.5. Clinical Implications and Future Perspectives

#### 3.5.1. Clinical Implications of Cutaneous Dysbiosis and Pathogen Reservoirs

The findings of this review underscore a critical paradigm shift: the skin of patients with diabetes mellitus (DM) should not be viewed merely as a passive barrier subject to mechanical failure, but rather as an active, metabolically compromised ecosystem that predisposes individuals to diabetic foot syndrome (DFS). The observed ecological collapse—characterized by a loss of protective commensals and an overrepresentation of Gram-negative taxa and virulent *Staphylococcus aureus* strains—suggests that dysbiosis occurs as a continuum. A critical unresolved issue is identifying the exact inflection point at which microbial remodeling transitions from a physiological adaptation to the hyperglycemic environment to a definitive risk factor for ulceration.

Furthermore, the clonal similarity between *S. aureus* isolates recovered from intact skin and active ulcers suggests a potential role for the surrounding skin as a microbial reservoir. However, this observation does not establish the direction of transmission or demonstrate that preventive skin decontamination reduces ulceration or infection risk. Therefore, microbiome modulation and targeted decontamination of intact skin should be regarded as hypotheses for future research rather than established preventive recommendations in clinical practice.

#### 3.5.2. Overcoming Translational Barriers in Microbiome Research

While the therapeutic potential of probiotics, advanced delivery systems, and bacteriophages is immense, their integration into routine clinical practice is hindered by significant translational barriers. The current literature relies heavily on cross-sectional study designs and is characterized by methodological inconsistencies, particularly regarding sampling techniques (swabs vs. biopsies), sequencing platforms, and anatomical site standardization. Additionally, the vast majority of research remains bacteriocentric, largely neglecting the vital roles of fungi and viruses within the wound ecosystem. Addressing the mycobiome is of paramount clinical importance, as the dominance of fungal taxa such as Ascomycota has been independently associated with a 2.1-fold increase in healing duration, whereas commensal *Malassezia* species may actively support epidermal regeneration [30]. Furthermore, existing microbiome literature suffers from severe geographical and ethnic biases; expanding phylogenetic profiling to underrepresented demographic groups, such as Afro-Caribbean populations, is critical for uncovering distinct microbial signatures and addressing the high proportion of unclassified fungal lineages currently absent from standard reference databases [43].

To bridge the gap between bench and bedside, the field urgently requires robust, prospective longitudinal studies that track microbiome dynamics from DM diagnosis through DFS onset and healing. As demonstrated by foundational longitudinal cohorts, tracking the wound microbiome across multiple time points is essential to capture the abrupt ecological shifts associated with acute infection episodes, antibiotic exposure, and tissue repair.

Integrating next-generation metagenomic sequencing with functional metatranscriptomics and metaproteomics, alongside artificial intelligence (AI) and machine learning, will be essential to decipher complex multi-omic datasets [30,32]. Furthermore, AI-driven predictive modeling is increasingly enabling the development of “digital twins”—virtual wound models that allow clinicians to simulate and evaluate personalized microbiome reprogramming strategies in silico prior to in vivo application [31].

#### 3.5.3. Emerging Preclinical Strategies and Mechanistic Insights

While clinical evidence constitutes the core of current therapeutic frameworks, emerging preclinical models remain indispensable for deciphering the precise molecular mechanisms of dysbiosis-induced tissue damage and testing next-generation interventions. The experimental findings discussed in this subsection are presented as complementary mechanistic evidence and should not be interpreted as primary comparative clinical evidence. For instance, murine microbiota-transplantation models have demonstrated that the shift toward Gram-negative dominance actively drives early diabetic dermopathy. These Gram-negative pathogens release lipopolysaccharides (LPS) that activate host Toll-like receptor 4 (TLR4) and CD14 signaling pathways, driving substantial inflammatory cell infiltration even before an open ulcer forms [38].

Furthermore, to counteract systemic dysbiosis, preclinical studies evaluating targeted nutraceuticals, such as the bioactive flavonoid quercetin, have demonstrated profound remodeling of the gut microbiota. In diabetic rat models, quercetin enriched anti-inflammatory taxa (e.g., *Ligilactobacillus*) and depleted pro-inflammatory *Desulfovibrionia*, thereby stabilizing HIF1α and upregulating VEGF in the wound bed to accelerate closure [50]. Similarly, fecal microbiota transplantation (FMT) in animal models has yielded promising results by restoring intestinal barrier integrity (upregulating ZO-1 and Occludin) and accelerating wound closure via the IL-17A-mTOR-HIF1α signaling axis [32,48,50]. Incorporating these mechanistic insights into future human randomized controlled trials will be critical for advancing precision microbiome-targeted therapies.

### 3.6. Limitations of the Review

An important limitation of this structured narrative review concerns the scope of the literature search. Although the primary database search was designed to identify relevant evidence on the cutaneous microbiome in diabetes mellitus and diabetic foot disease, several emerging diagnostic and therapeutic topics were addressed primarily through relevant secondary literature. Consequently, the coverage of these specific fields may not be exhaustive, and some relevant primary studies may not have been identified. These sections should therefore be interpreted as an exploratory synthesis of emerging research areas and potential future applications rather than a systematic evaluation of the efficacy, safety, or clinical applicability of individual interventions.

Additional limitations should also be acknowledged. The primary electronic database search was restricted to publications from January 2020 to August 2026, although relevant earlier studies were identified through backward citation searching. Language restrictions may also have resulted in the exclusion of relevant publications. Furthermore, substantial heterogeneity in study designs, populations, anatomical sampling sites, microbiological collection procedures, and sequencing techniques limited the comparability of findings and precluded a meaningful quantitative synthesis. No formal study-level risk-of-bias assessment was performed, which represents an additional methodological limitation. Finally, as much of the available clinical evidence is observational or cross-sectional, the temporal relationship between microbiome alterations and diabetic foot ulceration remains uncertain, and causal conclusions cannot be established.

## 4. Conclusions

The available evidence suggests that diabetes mellitus and diabetic foot disease are associated with alterations in the cutaneous microbiome, although the nature and magnitude of these changes vary across populations, anatomical sites, and clinical settings. Changes in microbial composition, including the relative enrichment of opportunistic pathogens, may contribute to ulcer development and persistence; however, the predominantly observational evidence does not establish causality or the temporal sequence of these alterations. Molecular diagnostic approaches, such as mNGS, have expanded our understanding of microbial communities, although their clinical utility requires further validation. Microbiome-targeted interventions, including probiotics, nanotechnology-based platforms, CRISPR-Cas approaches, and bacteriophage therapy, represent promising areas of investigation, but current evidence remains insufficient to recommend their routine preventive or therapeutic use in diabetic foot disease.

To advance the clinical management of DFS, future research must address several critical imperatives:

1. Methodological Standardization: Establish universal protocols for sample collection, considering seasonal variations, demographic factors, and the inclusion of both healthy and pre-ulcerative diabetic groups.
2. Comprehensive Ecological Profiling: Shift from bacteriocentric models to multi-kingdom approaches that evaluate bacterial, fungal, and viral interactions within the wound bed.
3. Targeted Modulatory Therapies: Conduct large-scale randomized controlled trials to validate the long-term safety, dosing, and efficacy of advanced modulatory therapies and nanobiotechnology in restoring host-microbiota symbiosis.

Ultimately, the cutaneous microbiome may represent a valuable target for future risk stratification and preventive research in diabetes mellitus. Prospective longitudinal studies are needed to determine whether microbial alterations precede ulceration, while appropriately controlled clinical trials are required to establish the safety and clinical efficacy of microbiome-based interventions before their routine implementation.

## Figures and Tables

**Table 1:** Intrinsic and environmental factors influencing the microbial niche of the human foot.

Category	Factor	Influence on the Microbiome and Cutaneous Environment
Intrinsic Factors	Skin thickness	Variations in epidermal thickness create distinct structural microenvironments and microbial habitats across different areas of the foot.
	Anatomical characteristics	Structural features, such as interdigital folds, arches, and pockets, create protected microenvironments that favor specific taxa.
	Sweat gland distribution	Variations in eccrine gland density directly affect local moisture and salinity levels, thereby influencing microbial growth.
	Skin pH levels	The physiological acidic mantle limits pathogen overgrowth; alkaline shifts facilitate colonization by opportunistic bacteria and fungi.
Environmental and Lifestyle Factors	Hygiene practices	Regular washing and soap use can transiently alter microbial composition by removing superficial microbial layers and modifying skin pH.
	Footwear choice	Occlusive footwear creates a warm, dark, and moist microenvironment, drastically shifting the ecosystem to favor anaerobic microorganisms and fungal growth.
	Lifestyle and systemic factors	Diet, stress, metabolic conditions (e.g., hyperglycemia), and immune status profoundly influence skin barrier integrity and microbiome composition and balance.

**Table 2:** Methodological characteristics and ecological shifts in studies comparing the skin microbiota of individuals with diabetes mellitus (DM) and healthy controls (HC).

Study (Author, Year)	Study Design and Stratification	Sample Size and Demographics	Sampling Protocol	Key Ecological Shifts and Findings
Redel et al. (2013) [16]	Cross-sectional (HC vs. DM)	*n* = 60 HC: 30 (mean age: 59.2; M/F: 30/—) DM: 30 (mean age: 61.2; M/F: 30/—)	Sites: Plantar arch, dorsum, interdigital space, forearm. Technique: Swab (rubbed 30 times, duplicated).	Reduced overall abundance of the genus *Staphylococcus* in individuals with DM, but significantly greater presence of highly virulent *S. aureus* strains.
Gardiner et al. (2017) [18]	Longitudinal observational study (10 weeks) (HC vs. DM)	*n* = 16 HC: 8 (mean age: 62.8; M/F: 2/6) DM: 8 (mean age: 68.9; M/F: 5/3)	Site: Plantar arch. Technique: Moist swab (firm rubbing for 30 s).	Significantly lower microbial diversity on DM skin (435 OTUs) than on HC skin (998.5 OTUs), with a trend toward lower stability. *Kocuria* and *Micrococcus* were detected exclusively on HC skin.
Han et al. (2020) [24]	Cross-sectional (HC vs. DM), with a focus on the mycobiome	*n* = 35 HC: 18 (mean age: 62.9; M/F: 10/8) DM: 17 (mean age: 61.8; M/F: 10/7)	Sites: Plantar forefoot and interdigital space. Technique: Wet swab (50 passes).	Thirty-five differential fungal components were identified. Overall decreases in fungal richness and diversity were observed in individuals with DM, but significant enrichment of the dermatophyte *Trichophyton rubrum* was observed in DM samples.
Pang et al. (2020) [37]	Stratified by DM duration	*n* = 52 (13 per group) Group A: HC (age: 52.5) Group B: DM <2 years (age: 51.7) Group C: DM 5–8 years (age: 60.8) Group D: DM >10 years (age: 63.7)	Site: Left plantar arch. Technique: Swab (>100 passes in 30 s).	Alpha diversity gradually increased with increasing DM duration (from Group A to Group D). Dynamic shifts were observed in the predominant phyla (Firmicutes, Proteobacteria, Actinobacteria, and Bacteroidetes).
Zhang et al. (2023) [38]	Stratified by IWGDF ulceration risk	*n* = 90 (18 per group) HC and very low-, low-, moderate-, and high-risk groups (mean ages: 44.0–65.4)	Site: Plantar arch. Technique: Moist swab (>100 passes in 30 s).	An increase in species richness was observed in the highest-risk group. Progressive decreases in Gram-positive taxa (Firmicutes) and increases in Gram-negative taxa (Proteobacteria and Bacteroidotes) were observed with increasing ulceration risk.

(DM: diabetes mellitus; HC: healthy controls; M: male; F: female; OTUs: operational taxonomic units; IWGDF: International Working Group on the Diabetic Foot).

**Table 3:** Microbiome signatures and transitional dynamics: Methodological heterogeneity in studies comparing intact skin and DFS-associated ulcers [7,15,18,39,40,41,42,43,45].

Study (Author, Year) [Ref.]	Comparison Groups and Demographics	Anatomical Site and Sampling Protocol (Methodological Highlight/Bias)	Key Microbiome Signatures and Pathogen Profiles
Oates et al. (2012) [15]	Wound bed (*n* = 26) vs. contralateral intact skin (*n* = 26).	Site: Unspecified lower limb. Protocol: Moist swab (skin) vs. scalpel debridement (wound).	*S. aureus* was the most frequent pathogen. Wounds lacking a dominant pathogen exhibited a microbiota that was more ecologically similar to healthy skin.
Gardiner et al. (2017) [18]	Intact skin (*n* = 170) vs. wounds (*n* = 72).	Site: Plantar arch vs. wound. Protocol: Moist swab (intact skin) vs. dry swab (wound) and debridement.	Swab and deep debridement samples yielded similar taxonomic profiles. Ulcers were significantly enriched in Enterobacteriaceae, *Serratia*, and *Finegoldia*.
Min et al. (2020) [39]	Healthy skin (*n* = 20) vs. wound edge (*n* = 20) vs. wound bed (*n* = 20).	Site: Plantar aspect. Protocol: Wet swab (skin) vs. tissue biopsy (lesion & edge).	The wound bed exhibited a higher number of unique taxa. Elevated baseline abundance of Gram-positive anaerobic cocci (GPAC), especially *Peptoniphilus*, correlated with impaired healing.
Huang et al. (2022) [7]	Contralateral intact skin vs. active ulcers.	Site: Unspecified foot lesions. Protocol: Levine technique swabbing (skin/wound) and tissue biopsy (wound).	Marked ecological shift: Intact skin was dominated by Gram-positive/aerobic taxa (*Staphylococcus and Corynebacterium*); ulcers collapsed into Gram-negative/anaerobic dominance (*Prevotella and Bacteroides*).
Ferhaoui et al. (2023) [45]	Wound bed vs. healthy skin (*n* = 32 isolates).	Site: Unspecified. Protocol: Purulent sampling vs. swabbing (WGS analysis).	Intraindividual transmission: *S. aureus* (including MRSA) recovered from intact skin and active ulcers within the same patient shared the same clonal lineages and virulence genotypes.
Zhang et al. (2023) [40]	Intact skin (*n* = 26) vs. perilesional skin (*n* = 22) in individuals with DM.	Site: Plantar arch vs. perilesional boundary. Protocol: Moist swab (>100 passes).	Patients with active ulcers exhibited distinct taxonomic profiles, with heavy perilesional colonization by *Peptostreptococcus*, *Prevotella*, *Pseudomonas*, and Enterobacteriaceae.
Bruni et al. (2024) [41]	Intralesional area vs. perilesional skin (*n* = 10).	Site: Anatomical bias (mixed sampling from the sole, heel, tibia, and toes). Protocol: Undefined swabbing technique.	*Staphylococcus* showed higher relative abundance in the perilesional area (42%), whereas strict anaerobes such as *Finegoldia* were enriched intralesionally.
Ferhaoui et al. (2025) [42]	Contralateral healthy skin (*n* = 32) vs. wound bed (*n* = 32). Stratified by PEDIS.	Site: Unspecified foot lesions. Protocol: Swab (skin) vs. pus sample (wound). mNGS profiling.	Microbial shift strongly correlated with clinical severity. Severe anoxic lesions (PEDIS grade 4) exhibited a profound collapse in microbial diversity and were overwhelmingly dominated by Bacteroidetes.
Ojeh et al. (2026) [43]	DFU tissue vs. paired adjacent intact skin (n = 8 patients, all Afro-Caribbean males with type 2 DM).	Site: DFU wound bed and adjacent intact skin. Protocol: High-resolution phylogenetic profiling (including mycobiome analysis).	Fungal alpha diversity is significantly diminished in chronic DFUs. The study noted the concerning enrichment of multidrug-resistant fungi (*Candida duobushaemulonii*), highlighting geographical biases in previous datasets.

DM: diabetes mellitus; DFS: diabetic foot syndrome; OTU: operational taxonomic units; GPAC: gram-positive anaerobic cocci; MRSA: methicillin-resistant *Staphylococcus aureus*; WGS: whole-genome sequencing; mNGS: metagenomic next-generation sequencing; PEDIS: Perfusion, Extent, Depth, Infection, and Sensation grading system.

## Data Availability

No new primary data were generated in this structured narrative review. The evidence presented was obtained from previously published studies cited in the manuscript. The literature search strategies and the classification of selected publications are provided in Supplementary Materials S1 and S2, respectively.

## Supplementary Materials

The following supporting information can be downloaded at https://www.mdpi.com/article/10.3390/jcm15218578/s1: Supplementary Material S1: Detailed Literature Search Strategy and Filtering Criteria; Supplementary Materials S2: Categorization and Methodological Characteristics of Publications Included in the Narrative Synthesis (N = 32).

## Abbreviations

The following abbreviations are used in this manuscript:

16S rRNA	16S ribosomal RNA
AGEs	Advanced glycation end products
Agr	Accessory gene regulator
AhR/AHR	Aryl hydrocarbon receptor
AI	Artificial intelligence
AMPs	Antimicrobial peptides
ARGs	Antibiotic resistance genes
CcpA	Catabolite control protein A
CD11b	Cluster of differentiation 11b
CRISPR-Cas	Clustered regularly interspaced short palindromic repeats and CRISPR-associated proteins
CRP	C-reactive protein
DFS	Diabetic foot syndrome
DFU	Diabetic foot ulcer
DM	Diabetes mellitus
EDIN	Epidermal cell differentiation inhibitor
EPS	Extracellular polymeric substance
ER	Endoplasmic reticulum
Esp	Epidermal serine protease
FMT	Fecal microbiota transplantation
FnBPA	Fibronectin-binding protein A
GPAC	Gram-positive anaerobic cocci
HC	Healthy controls
HDAC	Histone deacetylase
HIF1α	Hypoxia-inducible factor 1-alpha
IFN	Interferon
IL-1RA	Interleukin-1 receptor antagonist
IL-1β	Interleukin-1 beta
IL-6	Interleukin-6
IWGDF	International Working Group on the Diabetic Foot
LPS	Lipopolysaccharides
MBTs	Microbiome-based therapies
MMP-9	Matrix metalloproteinase-9
mNGS	Metagenomic next-generation sequencing
MRSA	Methicillin-resistant *Staphylococcus aureus*
mTOR	Mammalian target of rapamycin
OTUs	Operational taxonomic units
P2	Perforin-2
PEDIS	Perfusion, Extent, Depth, Infection, and Sensation
PRISMA	Preferred Reporting Items for Systematic Reviews and Meta-Analyses
qPCR	Quantitative polymerase chain reaction
ROS	Reactive oxygen species
SANRA	Scale for the Assessment of Narrative Review Articles
SCFAs	Short-chain fatty acids
SCVs	Small colony variants
Spa	Staphylococcal protein A
SspB	Staphopain B
TCA	Tricarboxylic acid
TGF-β	Transforming growth factor-beta
TLR2	Toll-like receptor 2
TLR4	Toll-like receptor 4
TMAO	Trimethylamine-N-oxide
TNF-α	Tumor necrosis factor-alpha
VEGF	Vascular endothelial growth factor
WGS	Whole-genome sequencing
ZO-1	Zonula occludens-1
