# Supplementary material for: The Foot Skin Microbiome in Diabetes Mellitus: Transitional Dysbiosis, Ulceration, and Precision Microbiome-Based Therapeutics: A Narrative Review
Source: J Clin Med. 2026 Nov 3;15(21):8578. doi: 10.3390/jcm15218578 (PMC13764408; doi:10.3390/jcm15218578)
Supplement: Supplementary file 1 [file jcm-15-08578-s001.zip › jcm-4602133-supplementary.pdf]

Supplementary Material S1: Detailed Literature Search Strategy and Filtering Criteria

Table S1.1. General Search Parameters and Inclusion Filters.

Parameter Category	Specification
**Date of Search**	August 2026
**Publication Date Filter**	January 2020 – August 2026 (applied to the primary electronic database searches only).
**Target Concept Blocks**	(1) Microbiology (bacteria, microbiome, microbiota)<br>(2) Skin (skin, cutaneous, dermatology)<br>(3) Diabetes (diabetic, diabetes mellitus)<br>(4) Foot (foot, diabetic foot)
**Language Filter**	English and European languages
**Study Type / Subject Filter**	Human subjects or human tissue (exclusion of non-human/animal models)

Table S1.2. Database-Specific Search Strings and Results.

Database	Search String	Initial Hits	Hits After Filters
**PubMed / MEDLINE**	("bacteria"[Title/Abstract] OR "microbiome"[Title/Abstract] OR "microbiota"[Title/Abstract]) AND ("skin"[Title/Abstract] OR "cutaneous"[Title/Abstract] OR "dermatology"[Title/Abstract] OR "dermatologic"[Title/Abstract]) AND ("diabetes"[Title/Abstract] OR "diabetic"[Title/Abstract] OR "diabetes mellitus"[Title/Abstract]) AND ("foot"[Title/Abstract] OR "diabetic foot"[Title/Abstract])	170	51
**Scopus**	(TITLE-ABS-KEY(bacteria) OR TITLE-ABS-KEY(microbiome) OR TITLE-ABS-KEY(microbiota)) AND (TITLE-ABS-KEY(skin) OR TITLE-ABS-KEY(cutaneous) OR TITLE-ABS-KEY(dermatology) OR TITLE-ABS-KEY(dermatologic)) AND (TITLE-ABS-KEY(diabetes) OR TITLE-ABS-KEY(diabetic) OR TITLE-ABS-KEY("diabetes mellitus")) AND (TITLE-ABS-KEY(foot) OR TITLE-ABS-KEY("diabetic foot"))	781	257
**Web of Science (WoS)**	TS=(bacteria OR microbiome OR microbiota) AND TS=(skin OR cutaneous OR dermatology OR dermatologic) AND TS=(diabetes OR diabetic OR "diabetes mellitus") AND TS=(foot OR "diabetic foot")	1,429	309 *

* Note: Due to database structure, specific WoS filters were applied during the search process: Document type = Articles; Main concepts = Human Medical Sciences, Dermatology, Podiatry, and Human Medicine.

In addition to the date-restricted electronic database searches, the reference lists of relevant publications were examined to identify foundational studies. Six additional publications published before 2020 were selected through this process based on their relevance to the methodological and clinical objectives of the review. These publications are included in Supplementary Material S2 and correspond to the six records identified through citation searching in Figure 1 of the main manuscript.

Scope of Complementary Topics

The database search strategies presented in Table S1.2 were developed to address the central objective of the review: the cutaneous microbiome in diabetes mellitus and diabetic foot disease. No additional systematic searches specifically targeting each emerging diagnostic or therapeutic approach were conducted. These complementary topics were incorporated into the narrative discussion through the analysis of relevant publications, including narrative and other literature reviews. Their coverage was intended to provide a thematic overview and was not designed to be exhaustive.

Supplementary Material S2: Categorization and Methodological Characteristics of Publications Included in the Narrative Synthesis (N = 32).

The following table summarizes the 32 selected publications contributing to the thematic narrative synthesis. These publications encompass different types of scientific evidence, including original research articles, methodological studies, and narrative or other literature reviews. Articles are grouped according to the primary thematic domain addressed in the manuscript. Their inclusion in this table reflects their contribution to the narrative synthesis and should not be interpreted as indicating that all publications represent primary comparative clinical studies.

Supplementary Material S2 is intended to provide a thematic classification of the publications contributing to the narrative synthesis rather than a uniform study-level data extraction table. Additional information on study populations, sample sizes, comparator groups, sampling procedures, and main microbiological findings for selected primary comparative studies is provided in Tables 2 and 3 of the main manuscript. Relevant methodological limitations are discussed in Section 3.3.3.

Primary Thematic Domain	Study (Author, Year)	Study Design / Article Type	Reference Number
1. Diagnostic Techniques and Predictive Biomarkers	Yu et al. (2026)	Mini Review (AI & Dysbiosis)	[47]
	Wang et al. (2023)	Narrative Review	[18]
	Sun et al. (2022)	Narrative Review	[25]
	Huang et al. (2022)	Observational Study	[7]
	Liu et al. (2020)	Narrative Review	[16]
	Yang et al. (2016)	Bioinformatic Experiment	[19]
	Chakravorty et al. (2007)	Laboratory Experiment	[20]
2. Skin Microbiota: Diabetes Mellitus (DM) vs. Healthy Controls	Liu et al. (2024)	Narrative Review	[26]
	Gehrke et al. (2023)	Narrative Review	[27]
	White & Grice (2023)	Narrative Review	[49]
	Zhang et al. (2023)	Observational Study	[31]
	Pang et al. (2020)	Observational Study	[29]
	Han et al. (2020)	Observational Study	[21]

Primary Thematic Domain	Study (Author, Year)	Study Design / Article Type	Reference Number
	Min et al. (2020)	Observational Study	[38]
	Gardiner et al. (2017)	Observational Study	[28]
	Redel et al. (2013)	Observational Study	[30]
3. DFS Signatures: Healthy Skin vs. Active Ulcers	Ojeh et al. (2026)	Observational Study	[52]
	Ferhaoui et al. (2025)	Observational Study	[37]
	Bruni et al. (2024)	Observational Study	[36]
	Ferhaoui et al. (2023)	Observational Study	[32]
	Zhang et al. (2023)	Observational Study	[35]
	Oates et al. (2012)	Observational Study	[34]
	Gontcharova et al. (2010)	Observational Study	[33]
4. Microbiota-Targeted Therapies in DFS	Çelo et al. (2026)	Narrative Review	[51]
	Chen et al. (2026)	Narrative Review	[44]
	Kamal et al. (2026)	Narrative Review	[45]
	Molasy & Wrzosek (2026)	Narrative Review	[46]
	Huang et al. (2025)	Narrative Review	[39]
	Plumet et al. (2025)	Comprehensive Literature Search	[43]
	Shedaliya et al. (2025)	Narrative Review	[41]
	Madaan et al. (2024)	Narrative Review	[5]
	Srivastava et al. (2022)	Narrative Review	[40]
